# Effect of Adding Chestnut Inner Skin on Allergenic Protein, Antioxidant Properties, and Quality of Bread

**DOI:** 10.3390/molecules29040863

**Published:** 2024-02-15

**Authors:** Yoko Tsurunaga, Eishin Morita

**Affiliations:** 1 Faculty of Human Science, Shimane University, Matsue 690-8504, Japan; 2 Department of Dermatology, Faculty of Medicine, Shimane University, Izumo 693-8501, Japan; emorita@med.shimane-u.ac.jp

**Keywords:** bread, chestnut inner skin, hypoallergenic wheat, tannin, wheat-dependent exercise-induced anaphylaxis

## Abstract

Wheat-dependent, exercise-induced anaphylaxis has no fundamental cure and requires patients to refrain from wheat consumption or to rest after eating. Although hypoallergenic wheat production by enzymatic degradation or thioredoxin treatment has been investigated, challenges still exist in terms of labor and efficacy. We investigated a hypoallergenic wheat product manufacturing technology that takes advantage of the property of tannins to bind tightly to proteins. Commercially available bread wheat (BW) and hypoallergenic wheat (1BS-18 “Minaminokaori”, 1BS-18M) were used. Chestnut inner skin (CIS) was selected as a tannin material based on the screening of breads with added unused parts of persimmon and chestnut. Hypoallergenicity was evaluated using Western blotting. The effect of CIS addition on the antioxidative properties of bread was also measured. For both BW and 1BS-18M, CIS addition reduced the immunoreactivity of wheat allergens. Antioxidant activities increased with increasing CIS substitution. However, 10% CIS-substituted breads were substantially less puffy. Five percent CIS substitution was optimal for achieving low allergenicity, while maintaining bread quality. The strategy investigated herein can reduce allergies related to wheat bread consumption. In this study, the evaluation of hypoallergenicity was limited to instrumental analysis. In the future, we will evaluate hypoallergenicity through clinical trials in humans.

## 1. Introduction

Wheat consumption is one of the most common causes of IgE-mediated food allergies in adults. Most adult cases of IgE-mediated food allergies to wheat show a type of wheat-dependent, exercise-induced anaphylaxis (WDEIA). The elimination of wheat from the diet or avoidance of exercise and nonsteroidal anti-inflammatory drugs (NSAIDs) after ingesting wheat are recommended measures to avoid allergic reactions in patients with WDEIA; however, these recommendations affect the quality of life of the patients to a large extent [1].

Wheat proteins are classified into soluble albumin, salt-soluble globulin, alcohol-soluble gliadin, and insoluble glutenin [2,3]. Gliadins are classified into α-, β-, γ-, and ω-gliadins based on electrophoretic mobility, and glutenin subunits are divided into high- (4W = 67,000–88,000) and low-molecular-weight (MW = 32,000–35,000) subunits. The ω-gliadins are further classified into ω1-, ω2-, and ω5-gliadins according to their amino acid sequences. Notably, ω5-gliadin and high-molecular-weight glutenin have been identified as major and minor allergens in WDEIA, respectively [4]. Enzymolysis/ion exchanger deamidation, thioredoxin treatment, and wheat strains lacking allergen-coding genes have been used to obtain hypoallergenic wheat [5]. The wheat products obtained using these approaches substantially reduce the reactivity of serum IgE in patients with WDEIA; however, they are either found to be ineffective in some patient populations or low levels of IgE reactivity are still observed for these products [5].

Tannins bind strongly to proteins and have long been used for purposes such as the leavening (removing protein-based cloudiness) of wine [6]. Proline-rich proteins and polymers have a high tannin-binding capacity [7]. As gluten proteins composed of glutenin and gliadin contain high amounts of proline and glutamine [8], and because the IgE epitopes of ω5-gliadin and high-molecular-weight glutenin subunits contain proline, tannins are speculated to form a robust complex with gluten proteins, thereby resulting in the low allergenicity of the gluten proteins. Chestnut and persimmon are reported to contain high levels of tannins [6]. However, the peels or immature fruit of chestnut or persimmon are discarded during commercial processing.

This study aimed to investigate the effect of the addition of the parts of chestnut or persimmon that are discarded during processing on the allergenicity of bread made from commercially available wheat flour. Tannin material can be added to cookies prepared using light flour to make them hypoallergenic [9]. We hypothesized that bread, a processed wheat product like cookies, could be made hypoallergenic by adding tannin. However, as bread is prepared using strong flour, it has a high ω5-gliadin content, which is an allergen, and it is unclear whether tannins can have a hypoallergenic effect. In addition to commercially available bread wheat (BW), hypoallergenic wheat (1BS-18 “Minamino Kaori”, 1BS-18M) was studied. The synergistic effects of breeding and tannin addition on hypoallergenicity were clarified. As gluten is important for allowing bread to retain its quality, the effect of adding chestnut inner skin (CIS), which had the highest hypoallergenic effect, on bread appearance was also evaluated. Furthermore, tannins have excellent antioxidant properties and are considered to prevent heart disease and cancer [10]; therefore, the effect of CIS addition on the antioxidant properties of bread was also investigated.

## 2. Results

### 2.1. Effect of Tannins on the Immunoreactive Protein Content of Bread

**Figure 1:**
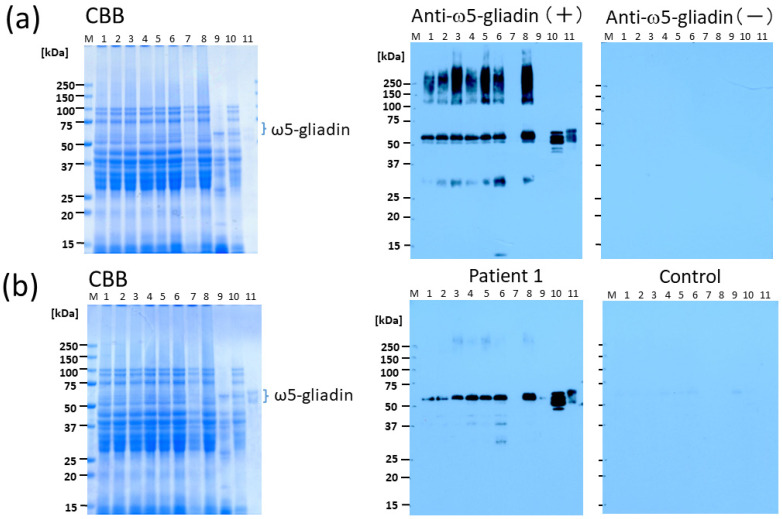
Detection of immunoreactivity in bread wheat (BW) bread using Western blotting with polyclonal rabbit anti-ω5-gliadin IgG Ab and serum obtained from a patient with WDEIA. (**a**) Bread samples (10 μg/lane), wheat flour samples (5 μg/lane), and purified ω5-gliadin (0.5 μg/lane) were electrophoresed, transferred, and blotted using anti-ω5-gliadin IgG Ab. Lane M, molecular weight marker; lane 1, bread without tannin material (control); lane 2, astringent persimmon peel bread; lane 3, non-astringent persimmon peel bread; lane 4, astringent persimmon fruit bread; lane 5, non-astringent persimmon fruit bread; lane 6, bread young persimmon fruit bread; lane 7, chestnut inner skin bread; lane 8, chestnut outer skin bread; lane 9, water-soluble fraction of wheat flour; lane 10, water-insoluble fraction of wheat flour; lane 11, purified ω5-gliadin. (**b**) Bread samples (10 μg/lane) and purified ω5-gliadin (0.5 μg/lane) were electrophoresed, transferred, and blotted using patient serum. Lane M, molecular weight marker; lane 1, bread without tannin material; lane 2, astringent persimmon peel bread; lane 3, non-astringent persimmon peel bread; lane 4, astringent persimmon fruit bread; lane 5, non-astringent persimmon fruit bread; lane 6, young persimmon fruit bread; lane 7, chestnut inner skin bread; lane 8, chestnut outer skin bread; lane 9, water-soluble fraction of wheat flour; lane 10, water-insoluble fraction of wheat flour; lane 11, purified ω5-gliadin. CBB, Coomassie brilliant blue; WDEIA, wheat-dependent exercise-induced anaphylaxis.

Coomassie brilliant blue (CBB) staining of sodium dodecyl sulphate–polyacrylamide gel electrophoresis (SDS-PAGE) gels showed that all bread samples (lanes 2–8) produced several protein bands in the molecular weight of 25–100 kDa that were similar to the protein patterns observed for the wheat-insoluble protein fraction (lane 10) and bread without tannin treatment (lane 1); however, the bands for bread treated with CIS (lane 7) were faint (Figure 1). Blotting with anti-ω5-gliadin IgG antibody revealed similar immunoreactive bands for the bread samples (lanes 2–6, and 8), with smear staining in the area above 100 kDa and band staining in the vicinity of 30 kDa and 55 kDa, whereas no staining was detected for the bread samples treated with CIS (lane 7) (Figure 1a). Blotting using serum from the patient with WDEIA revealed a 55 kDa band for the bread samples (lanes 2–6), except for the bread sample treated with CIS (lane 7), wherein no immunoreactive band was observed (Figure 1b). 

### 2.2. Effect of CIS Substitution on the Immunoreactivity of Bread Proteins

**Figure 2:**
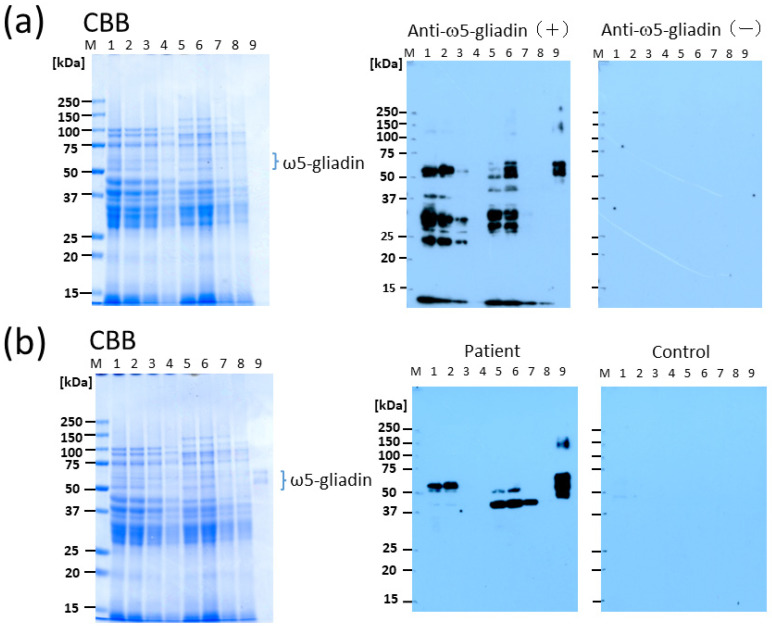
Detection of immunoreactivity in CIS bread using Western blotting with polyclonal rabbit anti-ω5-gliadin IgG Ab and sera obtained from patients with WDEIA. (**a**) Bread samples (10 μg/lane) and purified ω5-gliadin (0.25 μg/lane) were electrophoresed, transferred, and blotted using anti-ω5-gliadin IgG Ab. (**b**) Bread samples (10 μg/lane) and purified ω5-gliadin (0.25 μg/lane) were electrophoresed, transferred, and blotted using sera obtained from patient with WDEIA. Lane M, molecular weight marker; lane 1, BW bread without tannin material (control); lane 2, 3% CIS BW bread; lane 3, 5% CIS BW bread; lane 4, 10% CIS BW bread; lane 5, 1BS-18M bread without tannin material; lane 6, 3% CIS 1BS-18M bread; lane 7; 5% CIS 1BS-18M bread; lane 8, 10% CIS 1BS-18M bread; lane 9, purified ω5-gliadin. BW, bread wheat; CIS, chestnut inner skin; WDEIA, wheat-dependent exercise-induced anaphylaxis.

CBB staining of SDS-PAGE gels showed that the intensity of the bands decreased in a concentration-dependent manner with increasing percentages of CIS substitution (lanes 1–4 for BW bread and lanes 5–8 for 1BS-18M bread; Figure 2a), suggesting that the binding of tannin to gluten proteins inhibited CBB staining. Blotting with anti-ω5-gliadin IgG Ab showed that the immunoreactivity of the gluten proteins in the BW bread decreased with increasing percentages of CIS (lanes 1–4; Figure 2a). With 10% CIS substitution in the BW bread, no immunoreactivity was observed (lane 4). Similar reactivity patterns were observed for the 1BS-18M bread (lanes 5–8 Figure 2a). With 5% and 10% CIS substitutions in the 1BS-18M bread, no immunoreactivity was observed (lanes 7 and 8). IgE immunoblotting using serum from the patient with WDEIA revealed no immunoreactivity for the BW bread prepared using 5% (lane 3) and 10% CIS (lane 4), and none for the 1BS-18M bread prepared using 10% CIS (lane 8) (Figure 2b). 

### 2.3. Effect of CIS Substitution on the Soluble Tannin Content (STC), DPPH Radical-Scavenging Activity Value, and Hydrophilic Oxygen Radical Absorbance Capacity (H-ORAC) Values

**Figure 3:**
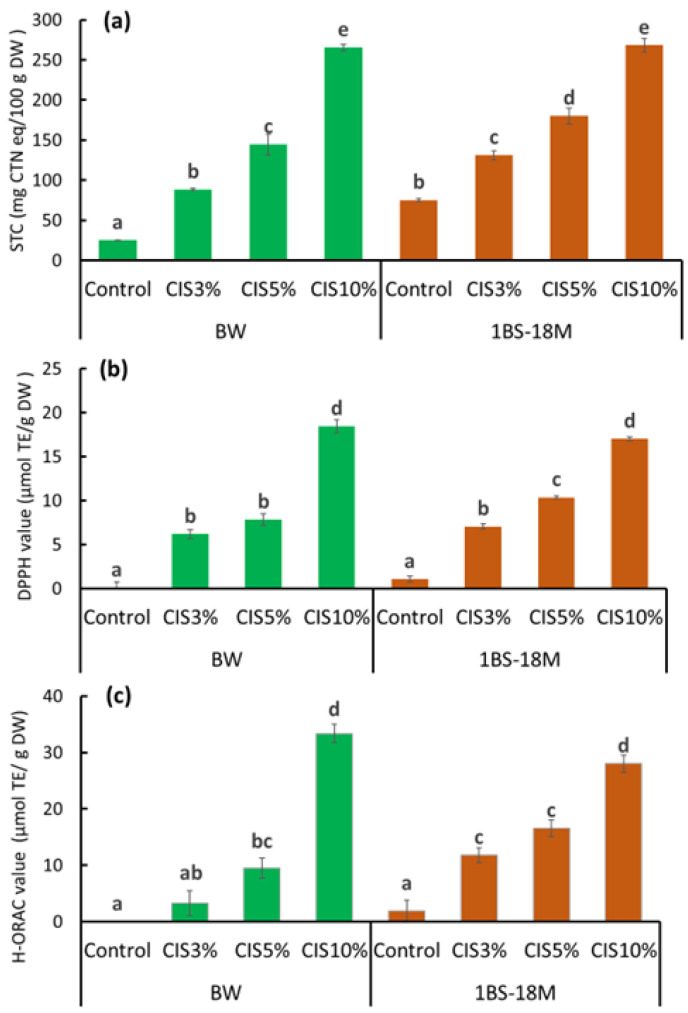
Effects of CIS substitution on STC (**a**), DPPH (**b**), and H-ORAC (**c**) values. The values are expressed as the standard reagent equivalent per unit dry weight. The results were analyzed using ANOVA followed by Tukey’s test for multiple comparisons. Data are expressed as mean ± SE (*n* = 6). The different letters indicate statistical differences (*p* < 0.05). ANOVA, analysis of variance; CIS, chestnut inner skin; DPPH value, DPPH radical-scavenging activity value; H-ORAC value, hydrophilic oxygen radical absorbance capacity value; SE, standard error; STC, soluble tannin content.

Figure 3 shows the effects of CIS substitution on the STC (Figure 3a), DPPH (Figure 3b), and H-ORAC (Figure 3c) values in the bread produced from BW or 1BS-18M. The STC, DPPH, and H-ORAC values increased markedly with 3% CIS substitution in both the BW and 1BS-18M bread samples. All three values tended to increase further with an increasing amount of CIS substitution and reached a maximum at 10% CIS substitution. 

### 2.4. Effect of CIS Substitution on the Appearance of Bread

**Figure 4:**
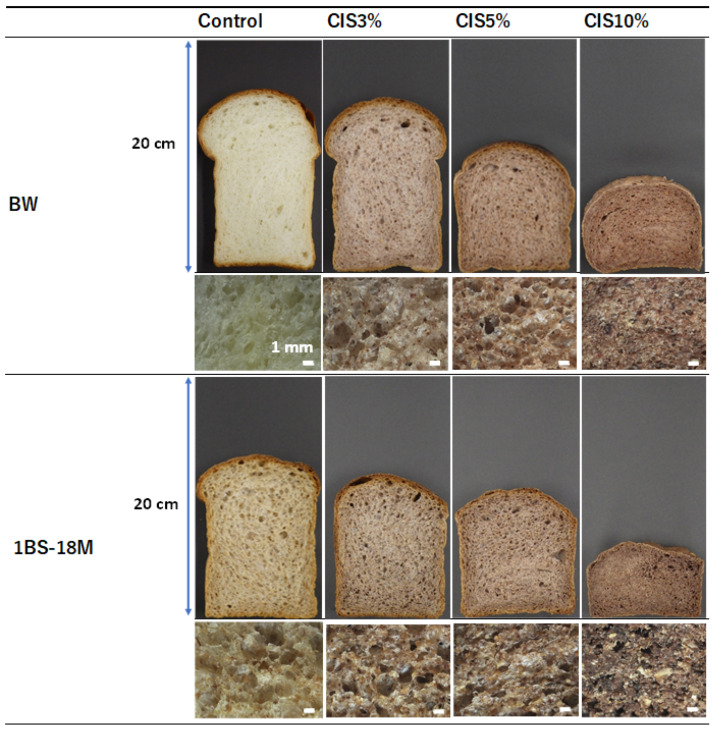
Evaluation of the effect of CIS substitution based on digital and stereomicroscopic images. White horizontal bars in stereomicroscopic images indicate 1 mm. CIS, chestnut inner skin.

Figure 4 shows the appearance and stereomicrographs of the breads produced from BW or 1BS-18M with 0–10% CIS substitution. A higher amount of CIS substitution revealed a smaller height and stronger brown coloration in both BW and 1BS-18M bread samples (upper images). In the stereomicroscopic images, although control BW bread showed numerous fine, raised bubbles of 12 mm diameter, the number of bubbles decreased with increasing CIS substitution (lower images). In particular, with 10% CIS substitution, the size of the bubbles was as small as 0.2 mm, and the number of bubbles was remarkably low. The control bread produced from 1BS-18M had larger and fewer bubbles than the control WB bread. In addition, as 1BS-18M was made from whole-wheat flour, numerous particles of 12 mm in diameter were observed, which appeared to be wheat hulls.

### 2.5. Effect of CIS Substitution on the Specific Volume of Bread

**Figure 5:**
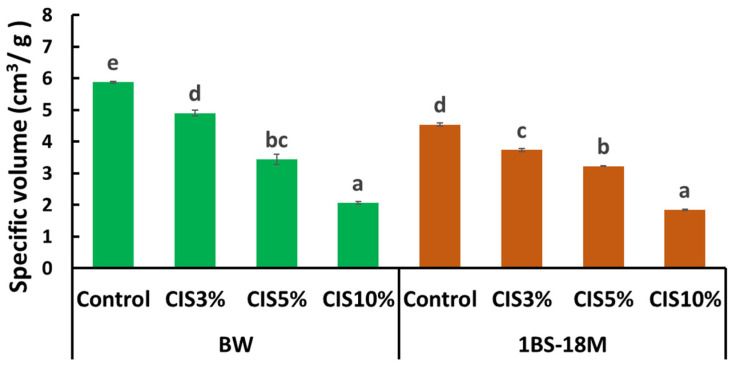
Effects of CIS substitution on specific volume. The results were analyzed using ANOVA followed by Tukey’s test for multiple comparisons. Data are expressed as mean ± SE (*n* = 3). The different letters indicate statistical differences (*p* < 0.05). ANOVA, analysis of variance; CIS, chestnut inner skin; SE, standard error.

The specific volume decreased substantially with increasing CIS amount in both BW and 1BS-18M breads. The specific volume of breads ranged from 2.1 ± 0.1 (10% CIS) to 5.9 ± 0.0 (control) cm^3^/g for BW and 1.8 ± 0.0 (10% CIS) to 4.5 ± 0.1 (control) cm^3^/g for 1BS-18M (Figure 5). 

## 3. Discussion

In this study, we demonstrated that CIS is an effective supplement in terms of reducing the allergenicity of bread, as determined by Western blotting using serum obtained from patients with WDEIA and anti-ω5-gliadin Ab. The tannin content of CIS has been reported to be remarkably higher than that of other plants [11,12]. Although the protein content of CIS-supplemented bread was lower than that of other bread preparations as determined using CBB staining after electrophoresis (Figure 1), the reduction did not seem to be substantial due to the low binding ability of CBB to the CIS-treated wheat proteins as a result of the interfering effect of tannins in the CIS. This finding suggests that tannin is the primary material that reduces the allergenicity of wheat proteins by binding to them, especially immunoglobulin-binding epitopes, in the process of bread preparation. The optimal substitution rate of CIS preparation of flour for achieving no allergenicity in making bread was 10%, wherein the net tannin concentration was approximately 2.3% DW of bread (Figure 2 and Figure 3). However, gluten formation appeared to be affected in this condition as the size of the bread decreased remarkably (Figure 4). It is likely that this method can be applied at a lower percentage substitution of CIS because, in Western blotting with anti-ω5-gliadin IgG Ab, only faint bands were observed for BW with 5% CIS (lane 3; Figure 2a) and the bands were completely lost for 1BS-18M at 5% CIS (lane 7; Figure 2a). In addition, in IgE immunoblotting using serum from patients with WDEIA, a 55-kDa band corresponding to ω5-gliadin was not observed at 5–10% CIS for either BW (lanes 3, 4; Figure 2b) or 1BS-18M (lanes 7, 8; Figure 2b); however, a faint band was observed at the area of 40 kDa for 1BS-18M with 5% CIS (lane 7; Figure 2b). It is inferred that the addition of tannins would be sufficiently effective with a 5% CIS substitution for patients with WDEIA, for which ω5-gliadin is the major causative agent of symptoms. 

Tannin is also known as an antioxidant substance [13]. Antioxidant properties are currently attracting substantial attention in terms of health functionality [12,13,14]. In addition, CIS reportedly exerts antibacterial effects [15] and lowers excessive fat in blood [16]; it is highly likely that these functions are also imparted to bread through CIS substitution. Notably, an increase in activity in the three assays (STC, DPPH, and H-ORAC) with increasing amounts of CIS substitution, particularly at 10% CIS, was observed. Bread supplemented with 10% CIS had a remarkably worse rise than that of other breads. The bubble structure of bread has a significant impact on texture and is an important factor in bread quality. The physical properties of starch glue during baking, the presence and distribution of sugars and fats, and other factors, but most importantly, gluten formation, are involved in bubble development. The formation of gluten requires the polymerization of gliadins and glutenins, which provide flexibility and elasticity to the dough [17]. As mentioned above, tannin binding to gluten proteins may have resulted in insufficient gluten formation and failure to multiply the gluten network in bread supplemented with 10% CIS. The specific volume of bread is one of the most important indicators of technical quality and markedly influences consumer choice [18]. The specific volume of commercial wheat bread varies between 3.5 and 5.5 cm^3^/g [18]. Most breads produced in this experiment were within or close to this range. However, with 10% CIS substitution, the specific volume was 2.1 ± 0.1 cm^3^/g for BW bread and 1.8 ± 0.0 cm^3^/g for 1BS-18M bread, which were remarkably lower than the minimum specific volume of 3.5 cm^3^/g for commercial bread reported by Monteiro et al. [18]. Therefore, bread with 10% CIS was considered unsuitable for sale.

A limitation of this study is that the safety of this hypoallergenic bread has not been confirmed, and whether the tannin-binding to the allergen protein is maintained after digestion in the gastrointestinal tract also remains unknown, although the binding ability of tannin is known to be tight even under proteolytic conditions. In addition, the taste and flavor of the bread supplemented with tannin remain to be evaluated.

In conclusion, we exploited the strong protein-binding properties of tannins to reduce allergens in BW and 1BS-18M using CIS, a byproduct of food processing. In particular, for the hypoallergenic 1BS-18M, CIS substitution may have an excellent additional hypoallergenic effect. Bread is a processed wheat product that requires a gluten network; thus, the decrease in gliadin content required to form gluten due to the addition of CIS is directly related to a decrease in quality. The strategy considered here aims to reduce allergies while preserving bread quality. We recommend that the CIS substitution be limited to 5% to achieve low allergenicity, while maintaining quality. The benefit of the method used in this study is the simplicity of adding tannin extract during the bread-making process. However, further experiments are needed to apply it practically in actual food processing scenarios. In this study, only instrumental analysis was performed, and no human sensory testing or clinical assessment for hypoallergenicity was conducted. In the future, we plan to conduct sensory and clinical tests on hypoallergenicity in humans. We also plan to study the detailed mechanism of hypoallergenicity caused by the addition of tannin materials. Although we limited our selection of materials to unused parts of persimmon and chestnut, we believe that the results of this study show the potential for the effective use of discarded agricultural products. 

## 4. Materials and Methods

### 4.1. Preparation of Persimmon and Chestnut Powders as Tannin Materials

Based on previous studies, persimmons and chestnuts, which have been found to have high tannin contents, were used as tannin materials [11,19,20]. Persimmons of the variety “Saijo” were collected at the Shimane Agricultural Technology Center (Izumo City, Shimane Prefecture, Japan). Young persimmon fruit were collected on 9 August 2021; mature fruit were collected on 13 November 2021, and the calyx and seeds were removed. Peel and pulp samples of mature fruits were prepared with and without astringency. Fruits and peels whose astringency was removed using dry ice treatment were defined as non-astringent persimmon fruits and non-astringent persimmon peels, respectively, whereas fruits whose astringency was not removed were defined as astringent persimmon fruits and astringent persimmon peels, respectively. To remove the astringency, 5 kg fruits were placed in 1 mm thick polyethylene bags filled with 50 g (1% by weight) of dry ice immediately after harvesting. All the samples were freeze-dried, powdered with an Oster Blender (Osaka Chemical Co., Ltd., Osaka, Japan), passed through a sieve (1 mm mesh), and sealed in aluminum-laminated plastic bags (Ramizip AL-16; Seisannipponsha Co., Ltd., Tokyo, Japan). Fruit of the chestnut variety, “Polotan”, were harvested in Hidaka City, Saitama Prefecture, Japan. The peel was separated into outer and inner skins, which were dried in a constant-temperature incubator (DN-61; Yamato Scientific Co., Ltd., Tokyo, Japan) at 60 °C for 12 h. The dried peels were crushed and sieved (1 mm mesh) to obtain chestnut outer skin (COS) and CIS powders. The dry powders were stored at −25 °C until they were used in bread production.

### 4.2. Wheat Flour

Two wheat varieties, commercial BW and 1BS-18 “Minaminokaori” (1BS-18M) [developed through repeated backcrossing between 1BS-18 “Hokushin” (a wheat line lacking *Gli-B1*, the gene encoding ω5-gliadin on the short arm of chromosome 1B) [21] and “Minaminokaori” (a wheat strain widely grown for bread production in Japan)] were used. BW flour refers to flour with the bran completely removed and 1BS-18M flour refers to whole-wheat flour. The composition of wheat flour BM was 354 Kcal energy, 13.0 g protein, 1.5 g fat, 72.0 g carbohydrate, and 0 g salt equivalent per 100 g. The composition of 1BS-18 was 333 Kcal energy, 12.8 g protein, 2.0 g fat, 69.4 g carbohydrate, and 0 g salt equivalent per 100 g. 

### 4.3. Bread Production

First, to identify promising tannin materials, we prepared bread prototypes using various parts of persimmons and chestnuts, which have been reported to have a high tannin content. The basic ingredients were 250 g wheat flour, 10 g unsalted butter, 17 g white sugar, 6 g skim milk, 5 g salt, 2.8 g dry yeast, and 180 g water. In the tannin addition group, 5% (12.5 g) of the flour weight was substituted with tannin material. The bread was baked using a fully automatic bakery (SD-BMT1001; Panasonic Corp., Tokyo, Japan). After baking, it was immediately removed from the mold and sliced into 2 cm thick slices. It was dried at 25 °C for 72 h, and then prepared for Western blotting. 

Then, to clarify the effects of the amount of tannin material (CIS) on the allergenicity and quality of bread, a bread production test was conducted using a kneader (PK-601; Nihon Kneader Corp., Kanagawa, Japan), for which the time and temperature could be set in each step of the production process. Preliminary experiments found that when the amount of CIS substitution was greater than 10%, the dough did not hold together and the bread could not be produced. Therefore, in this experiment, the amount of CIS substitution was set to 10% or less. The sponge dough method, wherein kneading is performed twice, was used. Specifically, 120 g of wheat flour, 6 g of dry yeast, 20 g of sugar, and 204 g of water were placed in the kneader and kneaded for 5 min and aged for 10 min (25 °C). Next, 180 g of wheat flour and 4 g of salt were added, and the dough was kneaded for 5 min. Then, 15 g of butter was added, and the dough was further kneaded for 10 min (25 °C). A convection heat oven (Convection Oven NE-CBS 2700; Panasonic Co., Ltd., Osaka, Japan) was used for the first fermentation at 35 °C for 30 min, using the steam function. Then, the dough was degassed for 20 s using the kneader, removed from the kneader, divided into two parts, and subjected to a second fermentation at 35 °C for 15 min. The dough was then lightly restrained to release gas, placed in a mold for one loaf (9.7 (D) × 19.8 (W) × 9.6 (H) cm), and fermented at 35 °C for 40 min. Next, the bread was baked at 160 °C for 10 min, 190 °C for 20 min, and 200 °C for 5 min. CIS flour was added in two batches: in the first batch, 3, 5, or 10% weight of 120 g wheat flour was substituted with tannin materials, and in the second batch, 3, 5, or 10% weight of 180 g wheat flour was substituted. After baking, the bread was immediately removed from the mold and placed in a polyethylene bag (thickness: 0.04 mm, size: 400 × 280 mm), with the top opened, and cooled at 25 °C for 24 h. Then, the samples were taken for analysis of the color tone, specific volume, and textural properties. For allergen evaluation and soluble tannin content (STC) determination, powdered samples were analyzed after drying at 25 °C for 72 h. Three breads were produced per treatment.

### 4.4. Western Blot Analysis

Western blotting was performed as previously described [9]. The ω5-Gliadin was purified from the water-insoluble fraction of commercially available wheat flour as described previously [4]. Samples were dissolved in a sample buffer and heated at 95 °C for 5 min. To determine the protein content in the samples, the proteins were extracted using the RC DC Protein Assay Kit (Bio-Rad Laboratories, Hercules, CA, USA) to remove β-mercaptoethanol. The protein concentration was determined following the Lowry method using the DC Protein Assay Kit (Bio-Rad Laboratories) [9]. The samples were separated in 12.5% acrylamide gels using SDS-PAGE. Proteins were visualized using Coomassie brilliant blue (CBB) staining. For immunoblotting, the proteins were electrophoretically transferred to a polyvinylidene difluoride membrane (Immobilon-*p*; Merck Millipore, Burlington, MA, USA) and reacted with polyclonal rabbit anti-ω5-gliadin IgG Ab [9]. The ω5-Gliadin bound to anti-ω5-gliadin IgG was visualized using ECL Prime Western blotting detection reagents (Amersham, Buckinghamshire, UK) after being reacted with horseradish peroxidase-conjugated donkey anti-rabbit IgG (GE Healthcare, Buckinghamshire, UK). IgE immunoblotting was performed using serum obtained from a patient with WDEIA. The ω5-gliadin-specific IgE serum level was 25.5 and <0.01 UA/mL for a patient with WDEIA and a control subject, respectively, as determined using ImmunoCAP (rTri a 19: ω5-gliadin) (Thermo Fisher Diagnostics, Waltham, MA, USA).

### 4.5. STC and Antioxidant Activity Assays

Folin–Ciocalteu reagent solution (2 N), 1,1-diphenyl-2-picrylhydrazine (DPPH, 95%) (powder), Trolox (97%), 2,2′-azobis (2-amidinopropane) dihydrochloride (AAPH, 95%) (powder), and ethanol solution (99.5%) were purchased from Wako Chemicals Ltd. (Osaka, Japan). Fluorescein sodium salt (1 mg/mL in pure water) was purchased from Sigma-Aldrich (St. Louis, MO, USA). Catechin (CTN) (>98%, powder) was purchased from Funakoshi Corporation (Tokyo, Japan). STC, DPPH, and hydrophilic oxygen radical absorbance capacity (H-ORAC) values of the bread samples were determined. Samples were extracted in 60% ethanol at 40 °C for 2 h while being subjected to shaking. The STC of the extracts was measured using the Folin–Ciocalteu method [9]. The STC was expressed as mg equivalent/100 g of dry matter using CTN as the standard (mg CTN eq/100 g DW). The antioxidant activity of the extracts was analyzed using the DPPH [22] and H-ORAC assays [23]. The DPPH and H-ORAC values were expressed in µmol Trolox equivalent/g of DW (µmol TE/g DW). For STC and all antioxidant activity assays, extraction was performed twice per treatment, and measurements were obtained in triplicate per extraction. 

### 4.6. Bread Appearance Examination

The breads were cut into 2 cm thick slices, and the cut surfaces were photographed using a digital camera (WG-40 W; Ricoh Co., Ltd., Tokyo, Japan). Stereomicroscopic images (DS-L3; Nikon Corp., Tokyo, Japan) were also acquired. 

### 4.7. Specific Volume

The volume of each loaf of bread before cutting was measured using the rapeseed displacement method (AACCI Method 10-05.01) [24] and divided by bread weight to obtain the specific volume. Measurements were performed three times per treatment. The results are expressed as the mean ± standard error (SE). 

### 4.8. Statistical Analysis 

Data were statistically analyzed using SPSS version 28.0 (SPSS Inc., Chicago, IL, USA). Results are expressed as mean ± SE. The data were analyzed using one-way analysis of variance, followed by Tukey’s test for multiple comparisons. Statistical significance was set at *p* < 0.05. 

## Data Availability

The data that support the findings of this study are available from the corresponding author upon reasonable request.

## Abbreviations

Ab: antibodies; BW, bread wheat; CBB; Coomassie brilliant blue; CIS, chestnut inner skin; CTN, catechin; DPPH, 1,1-diphenyl-2-picrylhydrazine; DW, dry weight; ELISA, enzyme-linked immunosorbent assay; H-ORAC, hydrophilic-oxygen radical absorbance capacity; SDS-PAGE, sodium dodecyl sulfate polyacrylamide gel electrophoresis; TE, Trolox equivalent; STC, soluble tannin content; WDEIA, wheat-dependent exercise-induced anaphylaxis; 1BS-18M, 1BS-18 “Minaminokaori”.
